# Applying the Life Cycle Assessment to the Use of Biochar from Vine Pruning Waste as an Additive in Mortar

**DOI:** 10.3390/ma18245573

**Published:** 2025-12-11

**Authors:** Jorge Los Santos-Ortega, Javier Ferreiro-Cabello, Esteban Fraile-García, Fátima Somovilla-Gómez

**Affiliations:** SCoDIP Group, Mechanical Engineering Department, University of La Rioja, 26004 La Rioja, Spain; javier.ferreiro@unirioja.es (J.F.-C.); estebanfraile@unirioja.es (E.F.-G.); fatima.somovilla@unirioja.es (F.S.-G.)

**Keywords:** biochar, mortar, vine shoots, life cycle assessment, sustainability

## Abstract

The agricultural industry and corresponding waste materials represent a potential research area for applying circular economy strategies. This research is a life cycle assessment of the addition of different mass percentages (0.47%W, 1.52%W and 2.27%W) of biochar made from vine pruning waste to mortar mixes. The research involves two scenarios. Scenario I is an attributional approach, aligned with the Environmental Product Declaration and Carbon Footprint standards. Scenario II takes a consequential approach, including biochar removals, as well as products and materials avoided as a result of the decision to start using this additive in mortar. The key findings differ substantially. For instance, under the impact category of Potential Global Warming compared to the reference mortar (without biochar additive) (0.58 kg CO_2_ eq), Scenario I yields a 56.85% increase in emissions (+2.21 kg CO_2_ eq) for the 1.52%W mix. By contrast, under Scenario II, the same alternative mixture yields an environmental benefit with a 76.83% decrease in emissions (−0.45 kg CO_2_ eq). This research highlights the environmental benefits of reusing agricultural waste specifically in the construction sector and provides an example of a circular economy study.

## 1. Introduction

The revalorisation of waste generated in various sectors, such as agriculture and industry, is being defined as an effective circular economy (CE) strategy with the aim of meeting sustainable development goals [1]. The construction sector is not exempt from improving its environmental and economic sustainability, justified by its significant influence on global Greenhouse Gas (GHG) emissions. An example of this is that the cement industry is responsible for around 8–9% of total GHG emissions [2]. Consequently, a new path is being opened up, as mentioned above, by attempting to transform this agricultural waste, which until recently ended its life cycle by being burned to obtain thermal energy or disposed of directly in landfills, which has a number of environmental impacts.

Waste from the agricultural sector is proving to be very interesting, justified by the economic, environmental and technical profits of its respective application to construction materials, such as mortar and concrete. It should be emphasised that this agricultural waste has an advantage over other types of waste in that it is associated with direct removals of biogenic CO_2_ [3], where its accounting and quantification can represent a considerable environmental advantage [4].

Another viable alternative for this agricultural waste is its transformation into biochar using a process called pyrolysis, in which biomass is thermally decomposed into solids, liquids and gases in an environment with no or only partial oxygen presence [5,6]. These vegetable charcoals can be used directly on mortar and concrete matrices to generate a wastewater filter element for the purpose of removing contaminants or heavy metals [7,8,9]. Another use for this charcoal is as a cementitious material, both as a substitute and as an additive [10,11]. An example of this is the research by Kiran et al., where replacements of 5% by mass of cement improve mechanical properties and durability [12].

Following this initial overview and the current context regarding the reintegration of agricultural and industrial residues back into the value chain—specifically within the construction sector, the main objective of this research emerges. The aim is to determine the environmental impact associated with using a biochar produced from vine-pruning residues as an additive, in each mass percentage (%W), within a mortar matrix. From this starting point, the study seeks to explore a potential application of this mixture, namely the development of mortar-based filters for wastewater generated by the wine industry itself. In doing so, the work aims to reproduce a specific case of circular economy in which a residue is generated, treated and subsequently reused within the same sector, moving away from the traditional approach in which biochar is employed as a partial cement replacement.

The production of this biochar was defined and tested in previous research by Los Santos-Ortega et al. [13], where manufacturing conditions of 450 °C and 3 h of carbonisation time resulted in a biochar with a fixed carbon content of 80.4%C, which implies a certain quality of biochar [14] with a biochar yield of 27.38%. This preliminary situation has opened a new gap in the field of research into the use of these biochars, where a new route has been defined to make this biochar applicable to certain construction materials such as mortar and concrete.

To this end, this research compiles experimental results at the environmental level on the feasibility of using biochar as an additive in mortar mixes. Using the life cycle assessment (LCA) methodology [15,16], the results will be evaluated for different dosage values and different impact categories. It should be noted that this research does not consider the technical behaviour, i.e., the definition of the mechanical properties (characteristic compressive strength, tensile strength, carbonation, etc.) of biochar as an additive, leaving its study for future research.

## 2. Methodology—Life Cycle Assessment

This section outlines the methodology required to create a life cycle assessment (LCA) for the use of biochar as an additive in mortars. The LCA is carried out according to the methodology set out in standards UNE-EN ISO 14040 [15] and UNE-EN ISO 14044 [16]. This study also used the UNE-EN ISO 15643 [17] and UNE-EN ISO 15804 [18] standards due to the fact that the material being assessed is widely used in the construction sector.

### 2.1. Objective and Scope

The main objective is to determine the environmental impacts of introducing an additive such as biochar made from vine pruning waste from the agricultural sector into a conventional mortar mix. This biochar is added in various mass percentages (%W).

### 2.2. Functional Unit

The Functional Unit (FU) proposed for this research is the manufacture of a mortar mix equivalent to a volume of 0.0032 m^3^ (approximately the amount required to produce two 10 × 10 cm mechanical compression test specimens for the standard [19]).

### 2.3. System Boundary

The next step involves determining the system boundary that help define the scope and objective of the LCA. The system boundary encompasses and circumscribes the individual processes that are included in the production of the product system and must therefore be studied in the LCA. The mortar material analysed in the LCA is a construction material widely used as classic masonry mortar to manufacture prefabricated elements such as perforated bricks, hollow bricks, etc. Therefore, its environmental analysis is affected by the UNE-EN 15804 [18] standard, which is used to issue Environmental Product Declarations (EPDs). UNE-EN 15804 [18] establishes a series of life cycle stages for a product in the construction sector, which are set out in Table 1.

**Figure 1:**
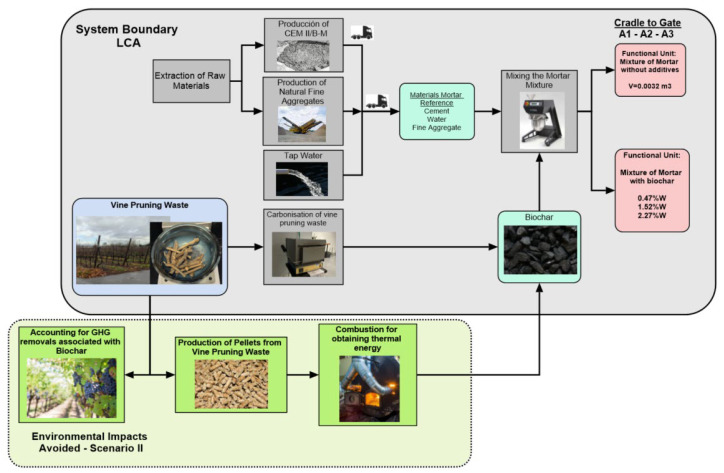
System boundary with a cradle-to-gate scope for manufacturing mortar with added biochar. The colour of the boxes indicates the type of item involved. Blue: waste; teal: manufactured material; red: functional unit; grey: unit process; green: avoided processes or products.

Although the process of generating an EPD is more complex and beyond the scope of this research, the decision was made to limit the system to a cradle-to-gate approach, which is equivalent to analysing processes A1: Raw Material Supply, A2: Transport to the Factory and A3: Material Manufacturing. This system boundary can be seen in Figure 1.

### 2.4. Accounting for Greenhouse Gas (GHG) Removals Associated with Biochar

A major debate surrounding the use of agricultural waste in construction materials concerns whether biochar associated with agricultural waste should be included in the LCA, i.e., whether this positive environmental impact should be included in the LCA. This is evident in the research conducted by Ee et al., which shows significant differences between the results of the LCA when this biochar is accounted for and when it is not [20]. Other previous research emphasises this fact and its strong influence on environmental viability results [21,22,23].

Therefore, a preliminary explanation is provided below that direct removal of CO_2_ emissions is associated with biochar derived from vine pruning waste (vine shoots). During its vegetative cycle, CO_2_ is sequestered directly by the vine plant for photosynthesis and then fixed as carbon (C) directly into the soil. Some studies assess this fixation at values of 9.12 tC per hectare of vine cultivation on average [24]. According to the study by Domenech et al. [25], vine cultivation is capable of generating a net sequestration of 6.26 tCO_2_ eq per hectare of cultivation (taking into account emissions associated with machinery, fertilisers and fuels), and, on average, one hectare of vine cultivation produces approximately 4.18 tonnes of shoots (this production is variable on an annual basis and depends on variables such as weather conditions). After this, it can be preliminarily established that 1 g of vine shoot is associated with a removal of 1.50 gCO_2_ eq according to the research conducted by Los Santos-Ortega et al. [13].

Following these guidelines, Table 2 shows the CO_2_ eq removals associated with the mass of biochar used as an additive in the mixes, where through the carbonisation process yield (η_Biochar Yield_) studied in previous research [13], the amount of vine pruning waste needed to produce biochar can be calculated and, consequently, its direct CO_2_ eq removals.

Strictly applying standards such as UNE-EN ISO 14067 [26], governing the calculation of a Product’s Carbon Footprint (PCF), establishes that Greenhouse Gas (GHG) removals are not taken into account in the LCA calculation. However, these removals can be documented in the PCF report for the user’s knowledge. Following the above observations on including the removals of biochar derived from an agricultural residue, two scenarios of results are proposed in this research (Scenario I and Scenario II). These scenarios are expected to provide insight into how differences in the LCA approach can affect environmental impact results.

**Scenario I—Not Accounting for Biochar**

The first scenario focuses on the Cut-Off attribution type LCA. The vine pruning waste is available in this approach without environmental impact within the boundary of the system proposed in the research (Figure 1). Only the environmental impacts associated with the unit processes of transforming this waste into biochar, such as the drying-carbonisation-crushing process in a muffle furnace, are applied to this waste. For this Scenario I, the GHG removals associated with the use of biochar and the products or processes avoided by using this biochar as an additive in mortars are not accounted for. This first scenario is more in line with the guidelines set out in regulations concerning the preparation of documents such as EPD and PCF.

**Scenario II—Accounting for Biochar + Avoided Products**

In the second scenario, the biochar present in the biochar itself will be accounted for in the LCA. In addition, a consequential approach is used in order to include those avoided products that are directly affected by the use of biochar as an additive in a mortar matrix within the system boundary (Figure 1). The changes that occur if a decision on the treatment of this waste is modified can be evaluated within this framework.

The first step is to objectively and logically define the main product obtained by transforming this vine pruning waste into biochar. Vine pruning waste is currently used as densified biomass for generating thermal energy in a conventional biomass boiler. Its calorific value varies depending on its moisture content (H%), with a lower calorific value (LCV) range of 4200–2310 kcal/kg for moisture percentages between 0 and 15%, respectively. The higher calorific value (HCV) is 4560 kcal/kg for zero moisture content, according to research by Fernández et al. [27], which qualifies vine pruning waste as a potential biofuel. However, before undergoing a combustion process to generate thermal energy, the biomass waste must be densified to obtain pellets, which adds another avoided unit process, namely the creation of pellets from vine pruning waste, which are assumed to have an HCV of 4200 kcal/kg after manufacture and for marketing. The results of energy and materials avoided in this Scenario II, are shown in Table 3, which lists the names of the processes selected in SimaPro.

As Ee et al. state, these strategies for accounting for saved energy are essential for demonstrating the environmental advantages that promote circular economy (CE) guidelines over traditional waste treatment and concrete production systems [20]. However, the modelling of Scenario II is not a universal reference, as there are multiple possible uses for these vine pruning residues, such as open field burning, shredding for direct degradation in the field. Therefore, the results obtained are not universally representative and may vary depending on how the avoided products have been modelled. To evaluate the influence of this situation, a sensitivity analysis has been carried out. In this analysis, the avoided product hypothesis from Scenario II is compared with the alternative in which vine pruning residues are subjected to direct open field burning, a practice that is currently very common in wineries. The results can be seen in Section 3.4.

### 2.5. Life Cycle Inventory

The Life Cycle Inventory (LCI) requires the compilation of all necessary data associated with energy consumption, consumables, etc., which are involved in the creation of the Functional Unit and according to the boundary of the system in question. When conducting an LCA, two different types of data can be identified. Primary data comes from direct measurements of variables involved in the study, such as energy consumption, quantities of materials, distances, etc. This type of primary data is very beneficial for the LCA because it provides less uncertainty due to its representativeness. Additionally, secondary data is obtained from external sources, such as previous research or similar LCAs. Both types of data were used in this research. The LCA modelling was carried out using SimaPro 9.2.0.2 software. Both the unit processes and all the data identified in the LCI have been taken from existing databases such as Ecoinvent v.3 [28].

#### 2.5.1. Primary Materials Used in Mortar (A1)

An initial explanation is provided for each material used in the mortar, detailing its main identified characteristics and the corresponding process associated with it in SimaPro. The proportions of these materials are shown in Table 4.

- Cement

The cement used to make the test specimens corresponds to CEM II/B-M (V-S-LL) 42.5 R, which means that this cement has a clinker content of between 65 and 79%, with the remaining elements being 21–35% siliceous fly ash (V), blast furnace slag (S) and limestone (LL). These are cements with a lower environmental impact. Its typical strength is 42.5 MPa at 28 days and has a density of 3150 kg/m^3^. Its main use is in the manufacture of prefabricated parts and concrete. See Table 5 to view the process involved in creating CEM II/B-M cement in SimaPro.

- Water

The water used for the test tubes corresponds to tap water (see Table 5), without special treatment. This water has a pH of 7.9 and a sulphate content of 590 ppm.

- Fine Aggregate

The fine aggregate comes from a gravel pit, with a grain size ranging from 0 to 6 mm, a density of 1634 kg/m^3^ and a fineness modulus of 3.16, making this fine aggregate equivalent to medium sand. See Table 5 to view the production process chosen in SimaPro for fine aggregate.

- Biochar

The additive material used in the mortar is biochar made from vine pruning waste, traditionally known as vine shoots. This biochar and its technical-environmental study are included in the previous research of Los Santos-Ortega et al. [13]. Broadly speaking, this biochar is produced in a muffle furnace that consumes low-voltage electrical energy (230 V) directly from the grid. The first step in manufacturing this biochar is to force-dry the vine pruning waste at 115 °C for 6 h to remove moisture. They are continuously carbonised at different temperatures, in this case 450 °C-3 h, due to the fact that these test conditions produced biochar with a fixed carbon content of 80.4% C, which indicates high-quality biochar [13]. The biochar production yield for these test conditions is η_BiocharYield_ = 27.8%, within a typical range of biochar yields [29]. Following this brief summary, the energy consumption recorded directly for the tests should be taken as a reference for this basic research, and, consequently, the direct measurement of the energy expenditure associated with revaluing this waste, such as its conversion to biochar.

Subsequently, it was recorded that producing 1 g of biochar requires 88 Wh of electrical energy (see Table 5 for the process selected in SimaPro). Therefore, Table 6 shows the respective energy consumption depending on the amount of biochar used in each mix. With the aim of incorporating in the environmental results section a sensitivity analysis (see Section 3.4) to evaluate how different biochar production conditions may influence the environmental impact, Table A1 is presented. This table summarises findings from a previous study [13], in which the biochar yields (η_BiocharYield_) and fixed carbon contents (%C) were recorded under various combinations of carbonisation temperature and residence time. It should be emphasised that this energy consumptions associated with biochar production correspond to a small-scale or laboratory-level process; on an industrial production scale, these energy demands would not be as high.

Once the biochar has been produced, it undergoes a grinding process in a jar mill, namely the NORMATEST (Madrid, Spain) model 48-D0544. This biochar is granulated to a size of 1–5 mm for subsequent incorporation into the mixer. This device has a power rating of 370 W at 230 V. The grinding time was 2 min, which gives an energy consumption value associated with the grinding production process of 12.33 Wh.

#### 2.5.2. Transport (A2)

This unitary process refers to the action of transporting the various primary materials (cement and fine aggregate) from their origin/manufacturing site to the place where the mortar mixes are produced, which in this case is the University of La Rioja’s premises. The distances travelled by cement and fine aggregate vary greatly in this situation and in Spain as a whole, as they generally depend on the location of the mortar processing plant. For lack of more precise data, the research by Mel Fraga et al. [30] is used, which proposes various distances travelled by these materials in the specific case of Spain.

Mel Fraga et al. [30] observe that, in the case of fine aggregate, Spain’s geomorphology means that aggregate is abundant, so the distances between production sites (quarries, gravel pits, etc.) are not very long, ranging from 15 to 60 km. An average distance of 30 km is assumed for this study. Cement, on the other hand, travels considerably further, due to the fact that there are fewer production facilities. The research again suggests a transport range of between 50 and 400 km, with an average of 200 km assumed. Finally, the additive element, biochar, is assumed to travel zero distance, due to the fact that it is produced and consumed on the University of La Rioja’s premises.

Following this transport process, the decision was made to provide greater definition in order to reduce uncertainty. Therefore, cement and fine aggregates are generally transported by 16–32 tonne lorries that consume diesel in an internal combustion engine, and these lorries are currently around 10–20 years old. Therefore, the European pollution regulations that affect them at source are EURO 4, which is shown in the transport process chosen in SimaPro (see Table 5). The unit of measurement for the transport process is kilogram-kilometre (kgkm). For cement, the unit transport process shall be 160 kgkm and 120 kgkm for fine aggregate.

#### 2.5.3. Mortar Manufacture (A3)

The mortar mix was prepared in a ControlGroup (Barcelona, Spain) digital mortar mixer, model 65-L0502. According to the technical data sheet, this model has a nominal power of 370 W and a 230 V input. The mixing of the materials took 180 s (3 min), and the additives (biochar) were included in the mixing process. Therefore, it is assumed that the energy cost in this unit process is approximately 18.50 Wh. Energy coming directly from the national electricity grid, the process selected in SimaPro can be view in Table 5.

### 2.6. Life Cycle Impact Assessment

Before proceeding to interpret the results, the UNE EN ISO 14040 standard [15] sets out the life cycle assessment stage, which consists of converting all the elementary flows defined in the LCI into environmental impact values. These values are then calculated and combined into a so-called impact indicator for each of the various categories assessed by each corresponding methodology. This whole process is composed of two sub-processes which are the classification process which basically consists of assigning for each impact category which flows identified in the LCI affect each impact category. The next stage is characterisation, i.e., transforming the various quantities of flows classified by impact category into an impact value using characterisation factors.

Each environmental impact assessment methodology may incorporate different impact categories and consequently also their respective characterisation factors, and there are variations in results between assessing the same LCA with one methodology or another. It is also important to note that the choice of an impact assessment methodology must be consistent with the case study. In reference to construction materials such as mortar and concrete, it is quite common for these materials to be defined by EPDs, which are verified documents that communicate to the consumer or customer the environmental impact of a product, corresponding to Type III Ecolabels governed by UNE-ISO 14025 [31]. Therefore, it was decided to use recognised environmental assessment methodologies to generate the EPDs for a construction product, with a view to replicating the case study, which is a construction material with a new additive, namely biochar. This is the EPD2018 methodology, whose impact categories and units of measurement are listed in Table 7.

## 3. Results and Discussion

After explaining the LCA methodology used in the case study, the main results obtained from the research are interpreted and discussed. This is divided into several sections. The first outcome aims to evaluate which materials and processes contribute most to the environmental impact of the reference mortar. Subsequently, the rest of the section compares the results of Scenarios I and II through a sensitivity analysis and, finally, an economic analysis.

It should be noted that the results are primarily presented based on the functional unit defined in this study (0.0032 m^3^ of mortar). However, the results have also been extrapolated to a larger volume of 1 m^3^. Accordingly, the relative increases or decreases in environmental impacts across the assessed impact categories remain effectively the same when scaled to 1 m^3^, due to the linear relationship between material quantities, mix proportions in the unit processes and the resulting environmental impacts. These outcomes are presented in detail in the following subsections.

### 3.1. Reference Mortar Without Biochar Added

**Figure 2:**
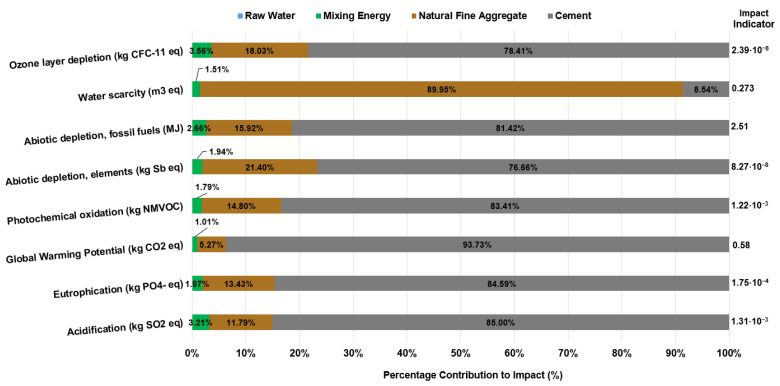
Environmental impacts of mortar without additives according to its primary materials.

This first result, although it may seem very obvious, is fundamental to understanding how any additive can affect mortar mixes and what strategies in terms of environmental results can be defined as future lines of research. Figure 2 shows the x-axis representing the percentage contribution (%) of each material in the mortar mix that directly affects that category of impact assessed. The right-hand column shows the indicator value for each impact category, i.e., the calculation of all environmental impacts (see also Table A2).

A first general observation shows that the main contributor to the generation of environmental impacts in mortar mixing and in the various impact categories is CEM II/B-M cement. This is followed, in second place, by natural fine aggregate (sand), then the unit process of mixing the materials. Finally, to a negligible extent, the environmental impact generated by the water used in the mixture. The main reason for arranging the environmental impacts on mortar in this way lies in the production process for generating CEM II/B-M cement, which involves high energy consumption, especially of fossil fuels or electricity from the grid. They are used directly as a fuel source in rotary kilns, where limestone (CaCO_3_) is decomposed into calcium oxide (CaO) and carbon dioxide (CO_2_) at around 1400–1500 °C, in the presence of secondary materials such as silica, iron and alumina to produce clinker or Ordinary Portland Cement (OPC).

This cement manufacturing process has a considerable impact, as shown in Figure 2, for impact categories such as Fossil Fuel Depletion 68.96% (2.04 MJ), Global Warming Potential 88.77% (0.539 kg CO_2_ eq) and other impact categories directly related to the use of traditional energy sources, Acidification 77.81% (1.11 × 10^−3^ kg SO_2_ eq) and Eutrophication 76.61% (1.48 × 10^−4^ kg PO_4_^−^ eq). Consequently, it follows that the higher the amount of clinker in the cement type, the greater the increase in environmental impacts. Therefore cements with high clinker content such as CEM I (95–100% by mass) have environmental impacts that are even 21.4% higher in certain impact categories such as Global Warming Potential than cements with lower clinker content such as CEM II (65–79% by mass) [32], which is a well-established sustainability strategy in the cement industry.

Taken from the functional unit of the reference mortar mix, the average impact percentage for cement material is 66.58%, followed by fine aggregate with 31.33% and finally the mixing and kneading process with an average impact value of 1.97%. For water, the impact is 0.12% for the various categories. These results show that if a cut-off rule were applied, which basically consists of establishing a base percentage, for example, 1%, those materials or unit processes whose impact contribution is less than the percentage established by the cut-off rule could be excluded from the LCA system boundary. This results in an effective way to save complexity in the process of elaborating the system boundary (Figure 1) and consequently the creation of the LCI.

### 3.2. Environmental Results—Scenario I

**Figure 3:**
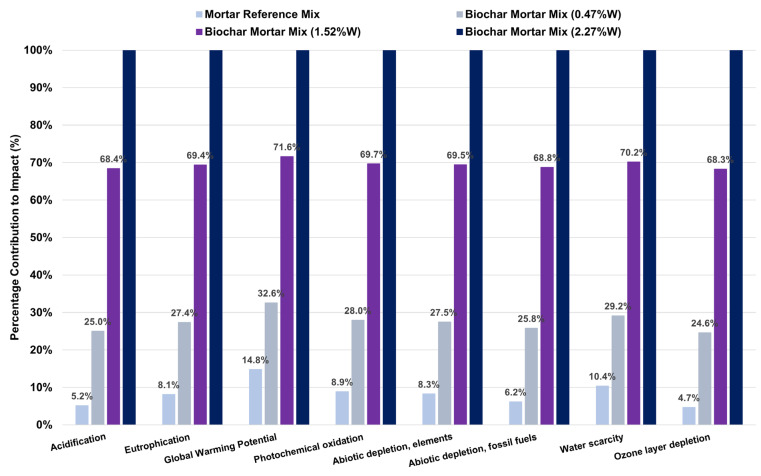
Environmental impacts Scenario I of the mixes as a function of their biochar content compared to the reference mix.

This first result (see Figure 3 and Table A3) explains the environmental comparison between the reference mortar mixes and those with a percentage of biochar additive added by mass for Scenario I, where the approach used is without incorporation of the biochar present in the biochar, as well as the processes/products avoided. As can be seen in general, as the use of biochar in the mix increases, the environmental impacts grow exponentially for all impact categories assessed in the EPD methodology.

The rationale is that biochar does not replace any primary element such as cement in the mix; therefore, there may be a certain environmental advantage in reducing the percentage by mass of the original CEM II/B-M. Secondly, the production of biochar from pruning waste has a fairly high environmental impact on the defined functional unit. These environmental burdens are mainly associated with the process of carbonising the vine shoot residues at 450 °C for three hours in a muffle furnace, whose energy source is the national electricity grid itself (see Table 6).

If we analyse the increases observed with respect to the reference mortar mix, for example, for Scenario I, in the Global Warming Potential impact category, it turns out that using 0.47%W of biochar leads to an increase of 17.83% (+0.7 kg CO_2_ eq), 1.52%W to an increase of 56.39% (+2.21 kg CO_2_ eq) and 2.27%W to an increase of 84.54% (+3.32 kg CO_2_ eq). These net increases may not be very high due to the size of the functional unit, with a volume of only 0.0032 m^3^. However, extrapolating to 1 m^3^ of mortar would result in very significant values. This is clearly presented in Table A5, where the results are evaluated for 1 m^3^ of mortar under the same biochar dosage conditions, but with an increased functional unit volume.

One of the strategies currently being used is to obtain the thermal energy in the carbonisation process from energy sources with lower environmental impact value where their respective CO_2_ eq emissions, for example, are lower. This is indeed the case, as at a larger, industrial production scale the associated energy consumptions for biochar production are not as high; otherwise, the process would be unfeasible not only from an environmental perspective but also economically. Industrial process optimisation, such as the introduction of heat recovery systems to improve efficiency, further reduces energy demand. To investigate this, a comparative study of CO_2_ eq emissions has been carried out based on different types of energy used to produce the biochar.

A practical example would be to compare the same amount of energy recorded in the carbonisation of biochar used in the research (see Table 6) but using two different technologies. The first of these energies is a muffle furnace that consumes energy from the grid, and the second is the same muffle furnace, but this one obtains energy from the combustion of biomass and natural gas. For this simulation, the final energy GHG emission factors provided by the Institute for Energy Diversification and Saving (IDAE in Spanish) are used [33] and their respective values are shown in Table 8. As can be seen, using energy sources with lower environmental impact in the manufacture of biochar would bring about a lower environmental burden on biochar and the results obtained with a cradle-to-gate approach would be lower than those seen in Figure 2.

The initial interpretation of Scenario I and its respective approach is that the addition of biochar as an additive in terms of mass percentage has a greater environmental impact than the reference mortar. However, although these results correspond to a productive scope, this does not imply that environmental assessments in later phases of use and maintenance do not show environmental advantages of this type of mortar with additives. Specifically, the environmental advantage of using waste from industrial or agricultural sectors is more visible in the medium to long term [32]. This is justified because these mortars with bio-agricultural waste often have improved physical and mechanical properties, such as improved thermal conductivity (λ) reflecting a decrease of up to 67% [11], which makes them more efficient when used as insulation in buildings. This results in improved energy efficiency and, consequently, a reduced environmental impact. Alternatively, there may be an improvement in mechanical performance, according to research by Barbhuiya et al. In the specific case of biochar, its high surface area and porosity increase water absorption and therefore the moisture content within the mixture, which promotes greater durability, reducing shrinkage during the setting process and improving the appearance of cracks at an early stage, as well as increasing mechanical strength [10,29].

In later stages of mortar use and application in a building, the carbon sequestered by the biochar mortar could also be taken into account, as it acts as a CO_2_ capture and retention agent from the atmosphere in the chemical process of concrete and mortar carbonation. Where the alkaline compound calcium hydroxide Ca(OH)_2_ combines with CO_2_ present in the atmosphere to form calcium carbonate CaCO_3_ plus water H_2_O. Many studies confirm that carbon sequestration can be an interesting way of mitigating GHGs associated with the construction sector [10,11,12]. Therefore, although Scenario I has shown that the environmental profitability of manufacturing mortar with biochar as an additive in weight percentage (%W) is not ideal from a cradle-to-gate perspective and for the production processes developed for biochar, this does not mean that it will be excluded from subsequent assessments. Even when applying more sustainable technology in the manufacture of biochar, more satisfactory results can be obtained.

### 3.3. Environmental Results—Scenario II

**Figure 4:**
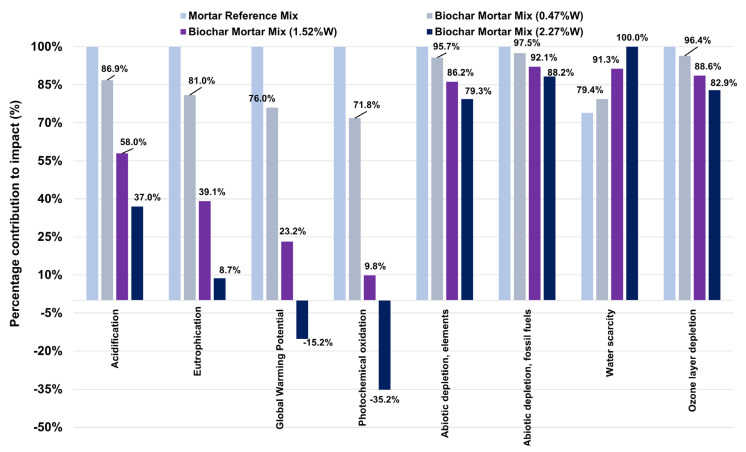
Scenario II environmental impacts of the mixes based on their biochar content compared to the reference mix.

Figure 4 shows the environmental results of Scenario II (see Table A4) and Table A6 shows the respective extrapolation to 1 m^3^ of mortar. The results for this scenario, compared with those obtained in Scenario I (see Figure 3), show a notable change in trend. In general, and for all impact categories except Water Scarcity, the various mortar mix alternatives incorporating a percentage of biochar in %W generate lower environmental impacts compared with the reference mortar.

Following on from the explanation of the GWP impact category, the reference mix produces the highest impact value with a value of 0.58 kg CO_2_ eq, whereas the mix with 0.47%W of biochar decreases by 24.01% (−0.14 kg CO_2_ eq), the mix with 1.52%W decreases by 76.83% (−0.44 kg CO_2_ eq) and finally, for the mix with 2.27%W, the decrease is 135.22% (−0.672 kg CO_2_ eq), which means that for this alternative, the impact indicator is negative with a value of −0.089 kg CO_2_ eq. Based on this initial observation, it is clear in Scenario II that accounting for biochar removals and environmental loads due to avoided processes and products gives the LCA results an environmental advantage when biochar is incorporated into mortar. This effect is consistent with current literature, for example, the research by Ee et al., where they evaluate a scenario of including biogenic CO_2_ eq in concrete, where they add biochar together with fly ash, partially replacing OPC. The results of their LCA for the inclusion of biochar imply significant differences for 1 m^3^ of concrete (f_ck_ = 30 MPa), specifically from 239 kg CO_2_ eq to values of around 96 kg CO_2_ eq [20]. Also, research by Rylko et al. associates a CO_2_ eq emissions credit to the use of biochar of −1.0 kg CO_2_ eq/kg biochar [34]. These situations considerably improve the LCA results, as also observed in the research by Wu et al., where replacing 5% of the amount of cement with biochar reduces CO_2_ eq emissions by 20.7% [35], as well as in the study by Shahmansouri et al., in which the use of up to 30% biochar as a cement replacement reduces CO_2_ eq by 76% [36].

Apart from the GWP impact category, other impact categories such as photochemical oxidation (−135.22%), ozone depletion (−17.07%), acidification (−63.01%) and eutrophication (−91.3%) show a reduction in their respective environmental impacts. These impact categories are limited as a result of modelling the combustion process of pellets in a biomass boiler to obtain thermal energy. Combustion processes generate various gases such as CO_2_ and NO_x_, as well as emissions of suspended particles and the creation of waste such as ash. Also, the pellet generation process, in its respective biomass densification process, the compacting machines’ main source of supply is electrical energy; therefore, their impacts directly affect the previously mentioned impact categories, as well as Fossil Fuel Depletion (−11.76%). Consequently, by modelling the avoidance of these effects, the environmental impacts of these impact categories are significantly reduced.

The only increase in the comparison is in the Water Scarcity impact category, where for mixes with a higher biochar content 2.27%W, the increase is 26.06% (+0.08 m^3^ of water), which is justified because as the mass content of vegetable carbon increases, the amount of electrical energy associated with this manufacturing process increases and, indirectly, the consumption of water used in the generation of electrical energy based on existing technologies.

**Figure 5:**
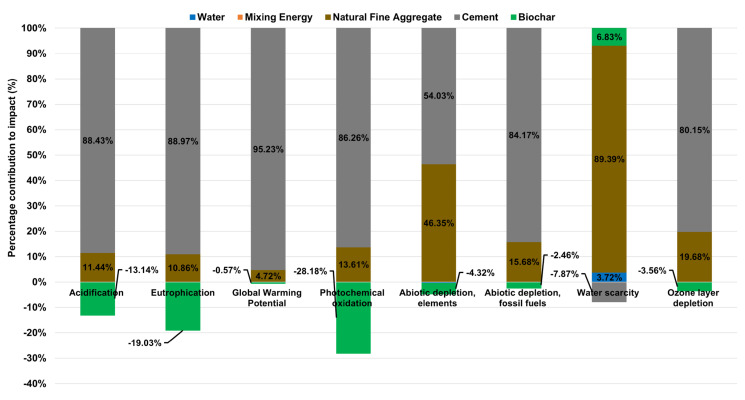
Environmental impact results for the mix with 0.47%W biochar as a function of its primary materials.

Figure 5 shows the contribution of environmental impacts as a function of material for the alternative of a mixture with 0.47%W of biochar. As can be seen for Scenario II, the results for cement and fine aggregate are very similar to those in Figure 2, with the exception of biochar. This clearly shows that in almost all impact categories, its percentage contribution is negative, with an average decrease of −10.18%, which implies that environmental impacts on the functional unit are being reduced.

Ultimately, in Scenario II, it is clear that the inclusion of biogenic removals in the LCA, as well as the justified modelling of avoided products and processes and, consequently, avoided environmental credits, give Scenario II very different results from those obtained in Scenario I.

In short, future lines of research arising from the current one can be divided into two situations. The first, based on Scenario I, where the use of biochar as an additive in %W by mass generates greater environmental loads, then resorts to the opposite situation, i.e., using it as a partial replacement for cement by mass. Research such as that conducted by Patel et al. found that a 2% substitution rate can increase compressive strength by around 18.95% [10]. It could be added and assumed that, apart from using less cement in the mix, this avoids the process of creating this material. Another line of research would be to quantify, in the early stages of setting and use of these biochar-based mortars and concretes, the net amount of CO_2_ eq sequestration and removal that occurs over a long period of use as a result of the carbonation process and to include this in the LCA study [37].

### 3.4. LCA Sensitivity Analysis

After explaining the results obtained in the study, it was decided to determine the level of uncertainty associated with these results. To this end, a sensitivity analysis was carried out on the various outcomes for Scenario I and Scenario II.

**Figure 6:**
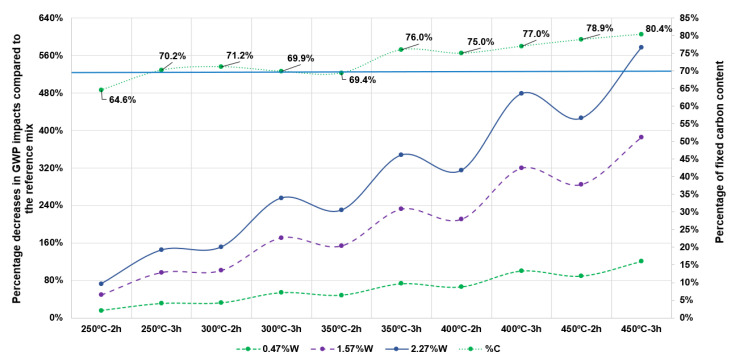
Sensitivity analysis for Scenario I in the GWP impact category, varying biochar manufacturing conditions.

First, the analysis focuses exclusively on the GWP impact category, examining the variations obtained in Scenario I when using the different types of biochar produced. These types of biochar differ in several parameters, including energy consumption, yield and, consequently, the percentage of fixed carbon content (see Table A1). The corresponding environmental results are illustrated in Figure 6.

As shown, the left y-axis represents the percentage increase in CO_2_-eq emissions relative to the reference mix, i.e., the alternative in which no biochar is used. The right y-axis corresponds to the fixed carbon content (%C) of each type of biochar. All modelled scenarios were calculated under the assumption that electricity is the energy source used for the carbonisation process.

It is evident that biochar production conditions involving higher carbonisation temperatures and longer residence times lead to greater percentage increases in impacts. This effect becomes less pronounced at lower dosage levels; for instance, when comparing the 2.27%W and 0.47%W dosages, the difference is substantial. Specifically, using 0.47%W of a high-quality biochar with a fixed carbon content of 80.4%C results in a 120.75% increase in GWP, whereas the same biochar at 2.27%W leads to a 577.06% increase. This biochar—characterised by its high fixed carbon content—was selected for the study due to its potential to enhance the properties of the mortar.

As a result of this sensitivity analysis, a key conclusion can be drawn: if the quality of the biochar incorporated into the mortar is limited to an intermediate range—for example, around 70% fixed carbon content (%C), which still represents a biochar of acceptable quality and applicability—a region can be identified in Figure 6 where the percentage increases in environmental impacts are not as pronounced relative to the reference mix.

For instance, under carbonisation conditions of 300–350 °C for 2–3 h and at lower dosage levels such as 0.47%W, the increases in GWP range from 32.06% to 72.99%. These values are relatively manageable and could be further reduced by using more sustainable energy sources, as shown in Section 3.2, or through industrial-scale production where process efficiencies are typically higher.

**Figure 7:**
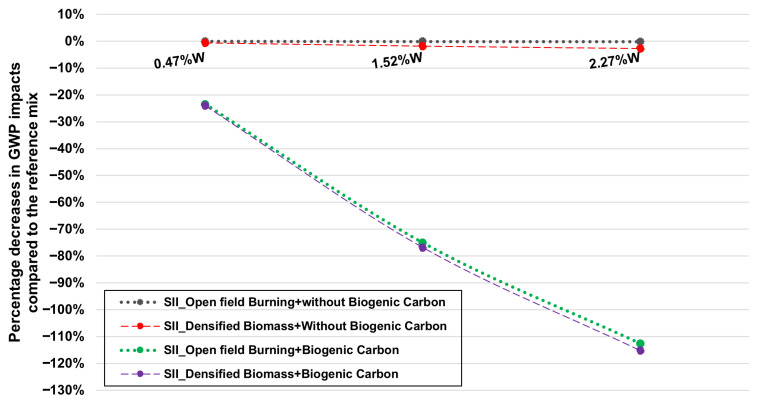
Sensitivity analysis for the GWP impact category as a function of the %W of biochar in the mortar.

The second sensitivity analysis corresponds to the environmental results of the LCA for Scenario II. The rationale for this analysis is that the avoided/substituted products defined in Scenario II are not the only possible treatment option for vine pruning residues, as explained in Section 2.4. Therefore, it was decided to assess whether there is an environmental benefit in using biochar in mortars under a scenario in which the vine pruning residues are prevented from being openly burned on-site at a winery. For the simulation and quantification of the avoided environmental credits, the process specified in Table 5 was selected. Additionally, within this first sensitivity analysis, the importance of including or excluding the accounting of CO_2_ removals associated with biochar use (see Section 2.4) was also studied, with the aim of understanding, under Scenario II, the main source of environmental benefit for the GWP category. These results are presented in Figure 7.

As can be observed, for each biochar dosage in the mortar mix, four distinct results are obtained. The upper results correspond to calculations where the carbon retained in the biochar itself (quantified in Section 2.4) is not included, whereas the two lower lines include it. Firstly, if we compare the two modelling approaches for the avoided products—namely, the production of densified biomass (pellets) and their subsequent combustion for heat generation, versus open field burning—the GWP impact category shows a slight environmental benefit for the first alternative of avoided products. For instance, for a 2.27%W biochar dosage, compared to the reference mix, the CO_2_ eq emission reductions are −2.7% for the pellets + thermal energy scenario, and only −0.7% for open field burning, which is a practically negligible difference. This is explained by the fact that pellet production involves unit processes with energy consumption from the grid, which increases its environmental impact value. One conclusion drawn is that the modelling of the avoided products associated with biochar derived from vine pruning residues is not of critical importance for the GWP impact category and its corresponding environmental benefit. Furthermore, it should be noted that these modelling approaches cover the vast majority of alternatives for the use, treatment, disposal, or valorisation of vine pruning residues.

On the other hand, if we consider whether to include the carbon sequestered in the biochar in the calculation, the conclusions differ. The main advantage in the GWP impact category is directly attributed to these CO_2_ eq removals. For example, for a 2.27%W biochar dosage in mortars, the GWP reduction is −115.2% compared to the reference mix, in contrast to the previously mentioned reduction of −2.7%.

**Figure 8:**
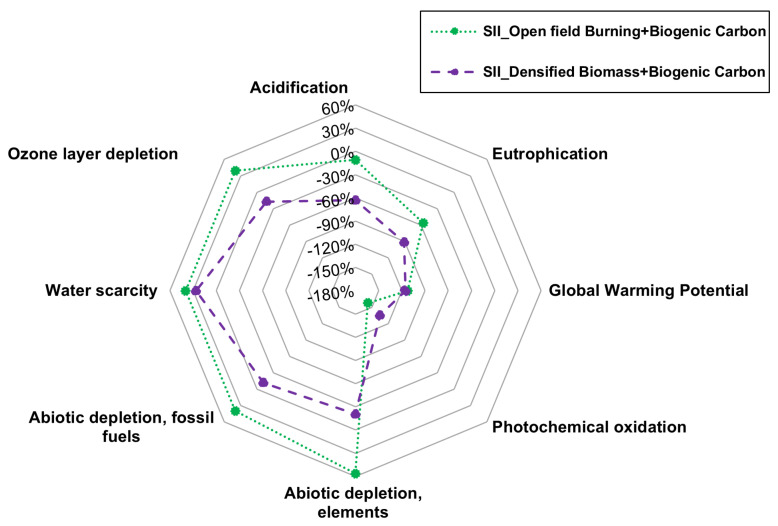
Sensitivity analysis for the remaining impact categories of the EPD methodology.

In conclusion, for this study, the inclusion of biogenic carbon in the biochar is essential for determining its environmental benefit under Scenario II and for the GWP category. The results of the sensitivity analysis for the GWP category are presented, while the differences for the remaining environmental impact categories are shown in Figure 8.

For the remaining impact categories, the situation changes substantially in the sensitivity analysis. For example, impact categories such as acidification (−11%; −63.01%), eutrophication (−56.15%; −91.30%), GWP (−112.6%; −115.25%) and photochemical oxidation (−157.54%; −135.22%), both avoided product alternatives result in reductions in environmental impacts, with slightly greater reductions observed when the avoided products are densified biomass (pellets) and their combustion for heat generation, compared to open-field burning. This indicates that, for these impact categories, both modelling approaches are positive.

Conversely, impact categories such as abiotic depletion elements (+56.30%; −20.69%), abiotic depletion fossil fuels (+39.83%; −11.76%), water scarcity (+39.62%; +26.06%) and ozone layer depletion (+39.38%; −17.07%) show significant differences between the two modelling approaches. For instance, open field burning of vine pruning residues in these categories results in percentage increases in environmental impacts compared to the reference mix, translating into higher environmental impact values.

The rationale is that, when analysing the unit process of biochar production, open-field burning does not contribute environmental impacts to these categories; the increases are solely due to electricity consumption in the muffle furnace. In contrast, the other avoided product alternative generates negative environmental impacts compared to the reference mix.

### 3.5. Economic Analysis of Mortar with Biochar

After discussing and explaining the environmental results, a fundamental aspect that significantly affects the decision to start using biochar as an additive has been left out of the research, namely the economic criteria. Therefore, this section aims to quantify in monetary value (unit of measurement in euros, EUR) the economic cost of manufacturing these mortars with biochar.

However, it should be noted that the price per functional unit (volume of mortar of 0.003 m^3^) is not very representative or significant in terms of magnitude, so it was decided to extrapolate the economic results to 1 m^3^ of mortar, along with the corresponding environmental impacts, as shown in Table A5. To calculate the unit prices of the materials, we refer to the research by Los Santos-Ortega et al. [38], which establishes a price of EUR 103/t for cement, EUR 9.5/t for fine aggregates and EUR 2/m^3^ for water. The price of biochar varies greatly depending on its previous production process and the origin of the biomass; however, over time, its economic value has fallen significantly [39].

**Figure 9:**
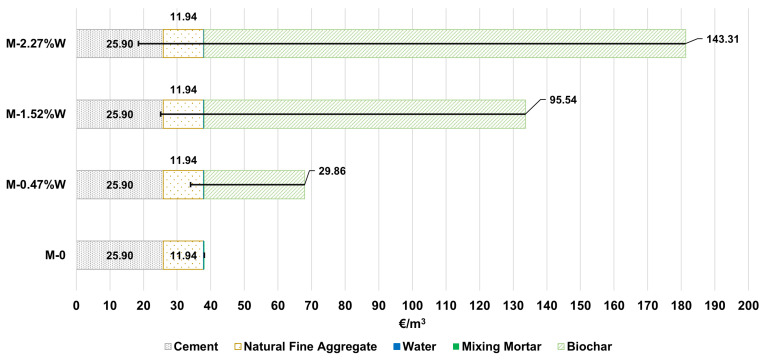
Economic results for 1 m^3^ of mortar as a function of biochar content, as well as a economic sensitivity analysis.

Compared to the price of cement, biochar is considerably more expensive, reflecting market conditions and the current real-world applications of biochar [10]. For this research, biochar from pruning waste has been chosen at an average price of EUR 4/kg according to a national market survey. The other economic cost to consider is electricity consumption during mixing operations. Mel Fraga et al. estimate that the process of mixlying 1 m^3^ of concrete consumes 1.61 kWh [30], which means that if the average price per kWh is EUR 0.14, the economic cost associated with mixing the concrete is EUR 0.23/m^3^. Table 9 shows the quantities of materials for 1 m^3^ of mortar and their unit prices. Figure 9 shows the resulting economic cost (EUR/m^3^) in cumulative form for the various mixes (1 m^3^).

The main results extracted from Figure 9 show that, in 1 m^3^ of mortar without biochar addition, the main economic cost per m^3^ is associated with cement, where with the dosages proposed in the research for cement (251.42 kg/m^3^), its respective cost accounts for 67.97% of the m^3^. Logically, with higher quantities of cement, its cost will increase considerably. On the other hand, the price of aggregates (1257 kg/m^3^) represents 31.33% of the economic cost. If both above materials are calculated, a total of 99.3% is established, with only 0.7% associated with the cost of mixing the mixture and the price of water use. These results remain constant as the quantities do not vary for the rest of the alternatives evaluated (0.47%W, 1.52%W, 2.27%W).

On the other hand, the incorporation of biochar as an additive linearly increases the monetary value, justified because its unit price is EUR 4/kg. As a result of increasing the percentage contribution by mass (%W) of the biochar additive, the price per m^3^ of mortar will increase. In the 2.27%W alternative, biochar has a cost of EUR 143.31/kg, which gives it a representative percentage of 79.05%, displacing cement to a percentage of 14.29% and fine aggregate to 6.6%. In short, this alternative is 375.86% more expensive than the reference mortar, rising from EUR 38.1/m^3^ to EUR 181.3/m^3^. This economic study is a partial cost analysis studying only the production stage (materials and energy) and is not a complete life cycle cost assessment of the biochar mortar.

Furthermore, it should be noted that biochar prices can fluctuate. In this study, the price analysed was for biochar sold to tertiary consumers, although prices may be lower than EUR 4/kg if supplied in large production volumes or in bulk. Despite this, it has been shown that adding biochar to mortar from cradle to gate reflects a substantial increase in economic prices, all within the scope of production. It is worth noting again that, if the use of these mortars results in mechanical and thermal advantages, it would be interesting to compare the economic savings that may exist in this situation with those of a reference mortar, which could offset economic profitability. Price limitations imposed by governments would also result in a strategy that promotes the use of this biochar in construction materials, as well as economic carbon removal programmes.

As an illustrative example, a simple quantification of the potential economic incentive for mortars containing biochar is presented, assuming payments for CO_2_ eq retention and sequestration were available. For this purpose, the average annual price of CO_2_ eq emission allowances for 2025 is used, considering this cost as the benefit obtained by avoiding emissions and sequestering CO_2_ eq. The 2025 average price is EUR 72.83/tCO_2_ eq [40]. Based on the CO_2_ eq retention values associated with biochar (see Section 2.4) and the amounts of biochar per m^3^ of mortar (see Table 9), the following reductions would be applied to the prices shown in Figure 9: 0.47%W (EUR −11.91/m^3^), 1.57%W (EUR −38.12/m^3^), and 2.27%W (EUR −57.18/m^3^). Additionally, for this economic sensitivity analysis, the biochar supply price is assumed to decrease to EUR 1/kg. The results are presented as error bars in Figure 9. As can be seen, the economic reductions are significant, and in some cases, the costs even fall below that of the reference mortar mix. This demonstrates that, although the production cost of mortar containing biochar is higher during the manufacturing phase, there are potential mechanisms to mitigate the economic cost, with additional reductions possible in later life cycle stages.

## 4. Conclusions

Key conclusions drawn from an environmental study of the use of biochar from vine pruning waste as an additive in mortar mixes are presented below.

Agricultural waste is becoming increasingly popular in the construction sector as additives or substitutes, but its environmental performance depends on the approach applied in the LCA. For Scenario II, the inclusion of biogenic GHG removals associated with biochar, as well as a consequential approach with the system expansion tool and including avoided processes and products, yields very different results from those explained for Scenario I with an attributional approach that is more oriented towards current regulations on Environmental Product Declarations and Product Carbon Footprints.

For instance, the disparity in the results obtained is such that, for the same alternative analysed, selecting the use of 1.52%W of biochar in the mortar mix and for an impact category such as Global Warming Potential, under Scenario I the impact indicator is 2.79 kg CO_2_ eq, and under Scenario II, it is 0.135 kg CO_2_ eq, representing a reduction of 95.16% between the percentage values. However, in Scenario II, the results are based on the determination of avoided burdens and should therefore be interpreted accordingly, rather than as a universally applicable outcome for all contexts

Regardless of the scenario (I or II), the definition of biochar intrinsic to waste is appropriate, where this research has stated that using additive quantities of around 25–120 g of biochar is associated with a removal of between 0.137 and 0.657 kg CO_2_ eq. Therefore, whether to include them or not in the LCA will depend on the study and scope, although it is advisable to indicate them.

Finally, the economic conclusions indicate that, under the current assumptions, the production of mortars with biochar currently involves a significant increase in economic costs. For the reference mortar alternative, the price is EUR 37.8/m^3^, while for the 0.47%W alternative, the price rises to EUR 181.3/m^3^, representing a percentage increase of 177.78%.

While the scope of the research was cradle-to-gate and showed that later-stage assessments may exist or justify environmental profitability for Scenario I, the lack of a more comprehensive vision or approach in the LCA on decision-making, such as incorporating waste from the agricultural sector into the construction sector, generates negative environmental results and a failure to observe reality, which may affect circular economy strategies.

## Figures and Tables

**Table 1:** Stages and processes associated with the environmental analysis of a building material according to UNE-EN 15804 [18]. The green tick indicates that this process is included in the LCA. The red cross indicates that this process has not been considered.

Phase	Process	Included in the LCA
Production	A1: Supply of raw materials	**✓**
	A2: Transport to the factory	**✓**
	A3: Manufacturing	**✓**
Construction	A4: Transport of the product	✕
	A5: Stages of the construction-installation process	✕
Product use	B1: Use	✕
	B2: Maintenance	✕
	B3: Repair	✕
	B4: Replacement	✕
	B5: Rehabilitation	✕
	B6: In-service energy use	✕
	B7: Operational water use	✕
End of Life	C1: Deconstruction and demolition	✕
	C2: Transport	✕
	C3: Waste treatment	✕
	C4: Waste disposal	✕

**Table 2:** CO_2_ eq removals associated with the use of biochar from vine pruning waste, depending on the dosage in mortar mixes.

Reference	Biochar Mass (g)	Direct Removal of CO_2_ eq (kg)
P1	25	−0.137
P2	80	−0.438
P3	120	−0.657

**Table 3:** Quantifying the energy and materials saved by using biochar as an additive in mortar.

Reference	Energy Avoided from Combustion (kWh)	Avoided Mass of Pellets from Manufacture (g)	Biochar CO_2_ Removal (kg)
P1	0.45	91.25	0.137
P2	1.43	292	0.438
P3	2.14	438	0.657

**Table 4:** LCI of the dosages for the mortar mix.

Reference	Fine Aggregate (g)	Cement (g)	Water (L)	Biochar (g)
P_REF	4.000	800	0.48	0
P1				25
P2				80
P3				120

**Table 5:** Life Cycle Inventory and the definition of its processes in SimaPro for the case study. The process nomenclature endings for Scenario I will be “Cut-Off”, while for Scenario II, the processes will follow the “Consequ” typology.

Process Description	Unit	Quantity	SimaPro Unit Process	Database
Basic Mortar Materials				
CEM II/B-M cement production	g	800	Cement, alternative constituents 31–35% {Europe without Switzerland} | production	Ecoinvent v.3
Transport of CEM II/B-M cement factory—University of La Rioja	kgkm	160	Transport, Freight, lorry 16–32 metric ton, Euro 4{RER}	
Gravel Pit Fine Aggregate Production	g	4.000	Sand {RoW} | gravel and quarry operation	
Transport of Gravel Pit Fine Aggregate—University of La Rioja	kgkm	120	Transport, Freight, lorry 16–32 metric ton, Euro 4{RER}	
Tap water	g	0.48	Tap water {RER} market group for |	
Electric power for mortar mixing	Wh	18.50	Electricity, low voltage {ES} | market for |	
Biochar Additive				
Biochar Production-University of La Rioja	p	Varies depending on the dosage of the specimen (see Table 6)	Electricity, low voltage {ES} | market for |	Ecoinvent v.3
Biochar Grinding in the Jar Mill	Wh	12.33	Electricity, low voltage {ES} | market for |	
Avoided Products—Scenario II				
Densification process of vine pruning residues (pellet production)	g	Varies depending on the dosage of the mix (see Table 3)	Wood pellet, measured as dry mass {RER} | market for wood pellet |	Ecoinvent v.3
Heat energy generation in a furnace	kWh		Heat, central or small-scale, other than natural gas {RoW} | heat production, softwood chips from forest, at furnace 50 kW.	
Open field burning of vine pruning residues			Waste wood, untreated {GLO} | treatment of waste wood, untreated, open burning | Conseq	

**Table 6:** Energy consumption associated with the quantities of biochar used in the mixes.

Reference	Amount of Biochar (g)	Manufacturing Energy Consumption for Biochar (kWh)
P1	25	2.19
P2	80	7.01
P3	120	10.52

**Table 7:** Environmental impact categories of the EPD methodology.

Impact Category	Unit
Acidification	kg SO_2_ eq
Eutrophication	kg PO_4_^−^ eq
Global Warming Potential	kg CO_2_ eq
Photochemical oxidation	kg NMVOC
Abiotic depletion, elements	kg Sb eq
Abiotic depletion, fossil fuels	MJ
Water Scarcity	m^3^ eq
Ozone Layer Depletion	kg CFC-11 eq

**Table 8:** CO_2_ eq emissions associated with producing biochar using two different energy sources. * The factor used for mainland electricity is 0.357 kg CO_2_ eq/kWh. ** The factor used for biomass is 0.018 kg CO_2_ eq/kWh. *** The factor used for natural gas is 0.252 kg CO_2_ eq/kWh.

Reference	Manufacturing Energy Consumption (kWh)	* Electrical Energy Source (kg CO_2_ eq)	** Biomass Source (kg CO_2_ eq)	*** Natural Gas Source (kg CO_2_ eq)
P1 (25 g biochar)	2.19	0.78	0.04	0.55
P2 (80 g biochar)	7.01	2.50	0.13	1.77
P3 (120 g biochar)	10.52	3.76	0.19	2.65

**Table 9:** Quantities of materials per 1 m^3^, as well as the unit prices of the materials.

Materials	Cement (kg)	Fine Aggregate (kg)	Water (L)	Biochar (kg)
1 m^3^ Reference	251.42	1257.09	150.85	0
1 m^3^ 0.47%W Biochar				7.86
1 m^3^ 1.52%W Biochar				25.14
1 m^3^ 2.27%W Biochar				37.71
Unit Price (EUR/tonne) except biochar (EUR/kg)	103	9.5	2	4

## Data Availability

The original contributions presented in this study are included in the article. Further inquiries can be directed to the corresponding author.

## Abbreviations

The following abbreviations are used in this manuscript:

LCA	Life Cycle Assessment
LCI	Life Cycle Inventory
FU	Functional Unit
PCF	Product Carbon Footprint
GHG	Greenhouses Emissions
CE	Circular Economy
EPD	Environmental Product Declaration
LCV	Low Calorific Value
HCV	High Calorific Value
kWh	Kilowatt hour
m3	Metric cubic

## Appendix A

**Table A1:** Results under different biochar production conditions as a function of carbonisation temperature and time.

Sample	Manufacturing Features	Biochar Yield (%)	%C	Manufacturing Energy Consumption for Biochar (Wh/g)
1	250 °C-2 h	62.33	64.60	11
2	250 °C-3 h	44.07	70.17	22
3	300 °C-2 h	36.24	71.24	23
4	300 °C-3 h	34.14	69.88	39
5	350 °C-2 h	32.01	69.44	35
6	350 °C-3 h	31.72	75.96	53
7	400 °C-2 h	28.52	75.00	48
8	400 °C-3 h	27.56	77.04	73
9	450 °C-2 h	25.09	78.89	65
10	450 °C-3 h	27.38	80.43	88

**Table A2:** Environmental results for the reference mortar mix.

Impact Category	Unit	Total	Tap Water	Mixing Energy	Natural Fine Aggregate	Cement
Acidification	kg SO_2_ eq	1.310 × 10^−3^	5.333 × 10^−10^	4.206 × 10^−5^	1.544 × 10^−4^	1.114 × 10^−3^
Eutrophication	kg PO_4_^−^ eq	1.749 × 10^−4^	6.584 × 10^−11^	3.454 × 10^−6^	2.350 × 10^−5^	1.480 × 10^−4^
Global Warming Potential	kg CO_2_ eq	5.756 × 10^−1^	1.077 × 10^−7^	5.812 × 10^−3^	3.031 × 10^−2^	5.395 × 10^−1^
Photochemical oxidation	kg NMVOC	1.220 × 10^−3^	2.604 × 10^−10^	2.185 × 10^−5^	1.805 × 10^−4^	1.017 × 10^−3^
Abiotic depletion, elements	kg Sb eq	8.275 × 10^−8^	3.540 × 10^−13^	1.604 × 10^−9^	1.771 × 10^−8^	6.343 × 10^−8^
Abiotic depletion, fossil fuels	MJ	2.507	1.224 × 10^−6^	6.662 × 10^−2^	3.991 × 10^−1^	2.041
Water scarcity	m^3^ eq	2.732 × 10^−1^	9.862 × 10^−6^	4.113 × 10^−3^	2.457 × 10^−1^	2.334 × 10^−2^
Ozone layer depletion	kg CFC-11 eq	2.389 × 10^−8^	1.316 × 10^−14^	8.503 × 10^−10^	4.307 × 10^−9^	1.874 × 10^−8^

**Table A3:** Environmental results for the various mortar alternatives based on their vegetable carbon content for Scenario I.

Impact Category	Unit	Mortar Reference Mix	Biochar Mortar Mix (0.47%W)	Biochar Mortar Mix (1.52%W)	Biochar Mortar Mix (2.27%W)
Acidification	kg SO_2_ eq	1.310 × 10^−3^	6.339 × 10^−3^	1.734 × 10^−2^	2.534 × 10^−2^
Eutrophication	kg PO_4_^−^ eq	1.749 × 10^−4^	5.880 × 10^−4^	1.492 × 10^−3^	2.149 × 10^−3^
Global Warming Potential	kg CO_2_ eq	5.756 × 10^−1^	1.271	2.791	3.897
Photochemical oxidation	kg NMVOC	1.220 × 10^−3^	3.832 × 10^−3^	9.548 × 10^−3^	1.370 × 10^−2^
Abiotic depletion, elements	kg Sb eq	8.275 × 10^−8^	2.746 × 10^−7^	6.943 × 10^−7^	9.995 × 10^−7^
Abiotic depletion, fossil fuels	MJ	2.507	10.47	27.90	40.58
Water scarcity	m^3^ eq	2.732 × 10^−1^	7.651 × 10^−1^	1.841	2.624
Ozone layer depletion	kg CFC-11 eq	2.389 × 10^−8^	1.256 × 10^−7^	3.480 × 10^−7^	5.098 × 10^−7^

**Table A4:** Environmental results for the various mortar alternatives based on their vegetable carbon content for Scenario II.

Impact Category	Unit	Mortar Reference Mix	Biochar Mortar Mix (0.47%W)	Biochar Mortar Mix (1.52%W)	Biochar Mortar Mix (2.27%W)
Acidification	kg SO_2_ eq	1.207 × 10^−3^	1.048 × 10^−3^	6.996 × 10^−4^	4.463 × 10^−4^
Eutrophication	kg PO_4_^−^ eq	2.099 × 10^−4^	1.700 × 10^−4^	8.209 × 10^−5^	1.826 × 10^−5^
Global Warming Potential	kg CO_2_ eq	5.839 × 10^−1^	4.437 × 10^−1^	1.353 × 10^−1^	−8.902 × 10^−2^
Photochemical oxidation	kg NMVOC	1.256 × 10^−3^	9.024 × 10^−4^	1.236 × 10^−4^	−4.425 × 10^−4^
Abiotic depletion, elements	kg Sb eq	3.409 × 10^−8^	3.262 × 10^−8^	2.938 × 10^−8^	2.704 × 10^−8^
Abiotic depletion, fossil fuels	MJ	2.417	2.357	2.227	2.133
Water scarcity	m^3^ eq	2.348 × 10^−1^	2.520 × 10^−1^	2.899 × 10^−1^	3.175 × 10^−1^
Ozone layer depletion	kg CFC-11 eq	2.142 × 10^−8^	2.066 × 10^−8^	1.898 × 10^−8^	1.777 × 10^−8^

**Table A5:** Environmental impact results for 1 m^3^ of mortar with biochar for Scenario I.

Impact Category	Unit	Mortar Reference Mix		Biochar Mortar Mix (0.47%W)		Biochar Mortar Mix (1.52%W)		Biochar Mortar Mix (2.27%W)	
		SI	%	SI	%	SI	%	SI	%
Acidification	kg SO_2_ eq	0.402	5.06%	1.975	24.85%	5.431	68.35%	7.946	100%
Eutrophication	kg PO_4_^−^ eq	0.054	8.0%	0.183	27.2%	0.467	69.3%	0.674	100%
Global warming potential	kg CO_2_ eq	179.61	14.7%	396.91	32.5%	874.65	71.6%	1222.17	100%
Photochemical oxidation	kg NMVOC	0.378	8.8%	1.195	27.8%	2.991	69.6%	4.297	100%
Abiotic depletion, elements	kg Sb eq	2.58 × 10^−5^	8.2%	8.57 × 10^−5^	27.3%	2.18 × 10^−4^	69.4%	3.14 × 10^−4^	100%
Abiotic depletion, fossil fuels	MJ	773.12	6.1%	3263.76	25.7%	8739.35	68.7%	12,722.47	100%
Water scarcity	m^3^ eq	88.02	10.7%	241.80	29.3%	579.88	70.2%	825.81	100%
Ozone layer depletion	kg CFC-11 eq	7.320 × 10^−6^	4.6%	3.911 × 10^−5^	24.5%	1.09 × 10^−4^	68.2%	1.598 × 10^−4^	100%

**Table A6:** Environmental impact results for 1 m^3^ of mortar with biochar for Scenario II.

Impact Category	Unit	Mortar Reference Mix		Biochar Mortar Mix (0.47%W)		Biochar Mortar Mix (1.52%W)		Biochar Mortar Mix (2.27%W)	
		SII	%	SII	%	SII	%	SII	%
Acidification	kg SO_2_ eq	0.379	100	0.329	86.9%	0.220	58.0%	0.140	37.0%
Eutrophication	kg PO_4_^−^ eq	0.066	100	0.053	80.9%	0.026	39.1%	0.006	8.6%
Global warming potential	kg CO_2_ eq	183.47	100	139.38	76.0%	42.39	23.1%	−28.14	−15.3%
Photochemical oxidation	kg NMVOC	0.395	100	0.283	71.8%	0.039	9.8%	−0.139	−35.3%
Abiotic depletion, elements	kg Sb eq	1.07 × 10^−5^	100	1.02 × 10^−5^	95.7%	9.2310^−6^	86.2%	8.49 × 10^−6^	79.3%
Abiotic depletion, fossil fuels	MJ	759.2	100	740.5	97.5%	699.5	92.1%	669.8	88.2%
Water scarcity	m^3^ eq	73.75	73.9	79.16	79.4%	91.07	91.3%	99.75	100.0%
Ozone layer depletion	kg CFC-11 eq	6.73 × 10^−6^	100	6.49 × 10^−6^	96.4%	5.96 × 10^−6^	88.6%	5.58 × 10^−6^	82.9%

## References

[1] United Nations Objetivos y Metas de Desarrollo Sostenible. https://www.un.org/sustainabledevelopment/es/sustainable-development-goals/.

[2] Miller S.A. (2018). Supplementary cementitious materials to mitigate greenhouse gas emissions from concrete: Can there be too much of a good thing?. J. Clean. Prod..

[3] Kharissova A.B., Kharissova O.V., Kharisov B.I., Méndez Y.P. (2024). Carbon negative footprint materials: A review. Nano-Struct. Nano Objects.

[4] Neve S., Barhemat D.R., Meng W., Bao Y., Sarkar D. (2023). Valorization of Vetiver Root Biochar in Eco-Friendly Reinforced Concrete: Mechanical, Economic, and Environmental Performance. Materials.

[5] Rosas J.G., Gómez N., Cara J., Ubalde J., Sort X., Sánchez M.E. (2015). Assessment of sustainable biochar production for carbon abatement from vineyard residues. J. Anal. Appl. Pyrolysis.

[6] Khater E.S., Bahnasawy A., Hamouda R., Sabahy A., Abbas W., Morsy O.M. (2024). Biochar production under different pyrolysis temperatures with different types of agricultural wastes. Sci. Rep..

[7] Muthu M., Sadowski L. (2024). Heavy metals removal in a graphene engineered concrete-based filter column. J. Build. Eng..

[8] Kim D.G., Ko S.O. (2023). The Pilot Study of a Dual-Media Filter Consisting of Mortar and Modified Zeolite for Removing Heavy Metals from Expressway Stormwater Runoff. Water.

[9] Praneeth S., Bordoloi S., Sarmah A.K. (2025). Low carbon biochar amended fly ash–cement building blocks: Assessment of metal leaching scenarios. Biochar.

[10] Patel R., Stobbs J., Acharya B. (2025). Study of biochar in cementitious materials for developing green concrete composites. Sci. Rep..

[11] Khan H., Zahid Z.B., Hussain F., Ahmad J., Khushnood R.A. (2025). Sustainable multifunctional biochar-based cementitious composites for carbon sequestration, energy storage, and smart infrastructure applications: A review. Case Stud. Constr. Mater..

[12] Kiran G.U., Nakkeeran G., Roy D., Alaneme G.U. (2025). Impact of biochar on strength, durability, and carbon sequestration in cement based materials. Discov. Sustain..

[13] Los Santos-Ortega J., Giró M.A.M., García E.F., Cabello J.F. (2024). Revalorisation of Vine Pruning Waste Through the Production of Zero CO2 Emission Charcoal. Appl. Sci..

[14] Andelini D., Cvitan D., Prelac M., Pasković I., Černe M., Nemet I., Major N., Goreta Ban S., Užila Z., Zubin Ferri T. (2023). Biochar from Grapevine-Pruning Residues Is Affected by Grapevine Rootstock and Pyrolysis Temperature. Sustainability.

[15] (2006). Environmental Management-Life Cycle Assessment. Principles and Framework. https://www.une.org/encuentra-tu-norma/busca-tu-norma/norma?c=N0038060.

[16] (2006). Environmental Management-Life Cycle Assessment—Requirements and Guidelines. https://www.une.org/encuentra-tu-norma/busca-tu-norma/norma?c=N0038059.

[17] (2021). Sustainability of Construction Works. Framework for Assessment of Buildings and Civil Engineering Works. https://www.une.org/encuentra-tu-norma/busca-tu-norma/norma?c=N0067460.

[18] (2020). Sustainability of Construction Works-Environmental Product Declarations-Core Rules for the Product Category of Construction Products.

[19] (2023). Testing Hardened Concrete-Part3: Compressive Strength of Test Specimens. https://www.une.org/encuentra-tu-norma/busca-tu-norma/norma?c=N0063272.

[20] Ee A.W., Chew S.J., Khoo H.H., Ng A.T.S., Kua H.W. (2025). Circular economy for the building industry: Life cycle assessment of biochar-enhanced concrete. Resour. Conserv. Recycl..

[21] Hoxha E., Passer A., Mendes Saade M.R., Trigaux D., Shuttleworth A., Pittau F., Allacker K., Habert G. (2020). Biogenic carbon in buildings: A critical overview of LCA methods. Build. Cities.

[22] Mekky K.M., Ibrahim M.G., Sharobim K., Fujii M., Nasr M. (2024). Evaluating environmental and economic benefits of using biochar in concrete: A life cycle assessment and multi-criteria decision-making framework. Case Stud. Constr. Mater..

[23] Peñaloza D., Erlandsson M., Falk A. (2016). Exploring the climate impact effects of increased use of bio-based materials in buildings. Constr. Build. Mater..

[24] Mesa I.F., Montserrat R.S., Loscos C.B., Vayreda J., Domínguez E.G.E., Aranda X., de Herralde Travería F. El cultivo de la vid como sumidero de carbono en La Rioja. Proceedings of the I Jornada del Grupo de Viticultura y Enología: Comunicaciones.

[25] Doménech J., Martínez M., Fernández M. (2010). La agricultura y el CO_2_: Los cultivos agrícolas captan más CO_2_ del que generan. El balance es positivo, 8.3 toneladas de media por hectárea al año. Cuad. Campo.

[26] (2019). Greenhouse Gases-Carbon Footprint of Products-Requirements and Guidelines for Quantification. https://www.une.org/encuentra-tu-norma/busca-tu-norma/norma?c=N0062518.

[27] Fernández J. (2006). Los residuos de las agroindustrias como biocombustibles sólidos (I). Vida Rural..

[28] Ecoinvent Database. https://support.ecoinvent.org/ecoinvent-version-3.0.

[29] Barbhuiya S., Das B.B., Kanavaris F. (2024). Biochar-concrete: A comprehensive review of properties, production and sustainability. Case Stud. Constr. Mater..

[30] Fraga J.M., Gochi A.C., López M.P.C. (2014). Sostenibilidad en la preparación y puesta en obra de hormigón en España: Análisis de consumo energético y emisiones de CO_2_. Proceedings of the 18th International Congress on Project Management and Engineering.

[31] (2010). Environmental Labels and Declarations—Type III Environmental Declarations—Principles and Procedures. https://www.une.org/encuentra-tu-norma/busca-tu-norma/norma?c=N0046196.

[32] Los Santos-Ortega J., García E.F., Cabello J.F. (2025). Environmental assessment of the use of ground olive stones in mortars. Reduction of CO_2_ emissions and production of sustainable mortars for buildings. Environ. Impact Assess. Rev..

[33] Factores de Emisión de CO_2_ y Coeficientes de Paso de a Energía Primaria de Diferentes Fuentes de Energía Final Consumidas en el Sector de Edificios en España. https://www.miteco.gob.es/content/dam/miteco/es/energia/files-1/Eficiencia/RITE/documentosreconocidosrite/Otros%20documentos/Factores_emision_CO2.pdf.

[34] Rylko I., Zarzychi R., Bobak L., Telega P., Didyk-Mucha D., Biatowiec A. (2025). Biochar as a Sustainable Component of Low-Emission Building Materials. Materials.

[35] Wu W., Lu Y., Qin Y., Xie J., Tan K. (2023). Life cycle assessment on CO_2_ emission for biochar concrete. J. Archit. Civ. Eng..

[36] Shahmansouri A.A., Zhang Z., Azarijafari H., Shi X. (2025). Biochar-amended high-strenght engineered cementitious composites. Cem. Concr. Compos..

[37] Jang D., Choi J., Bang J., Park J., Ham S.P., Yang B. (2025). Multifunctional role of biochar utilization in carbonation-cured cement mortar focusing on CO_2_ capture and thermal stability. J. Build. Eng..

[38] Los Santos-Ortega J., García E.F., Cabello J.F. (2024). Environmental and Economic Viability of Using Concrete Block Wastes from a Concrete Production Plant as Recycled Coarse Aggregates. Materials.

[39] Trapero J.R., Alcazar-Ruiz A., Dorado F., Sanchez-Silva L. (2025). Biochar price forecasting: A novel methodology for enhancing market stability and economic viability. J. Environ. Manag..

[40] SENDECO_2_ Prices CO_2_ Equivalent. https://www.sendeco2.com/es/precios-co2.

